# Association between coarse particulate matter and inflammatory and hemostatic markers in a cohort of midlife women

**DOI:** 10.1186/s12940-020-00663-1

**Published:** 2020-11-05

**Authors:** Emilie Davis, Brian Malig, Rachel Broadwin, Keita Ebisu, Rupa Basu, Ellen B. Gold, Lihong Qi, Carol A. Derby, Sung Kyun Park, Xiangmei (May) Wu

**Affiliations:** 1 Air and Climate Epidemiology Section, Office of Environmental Health Hazard Assessment, California Environmental Protection Agency, 1515 Clay Street, 16th Floor, Oakland, CA 94612 USA; 2 Department of Environmental Health Sciences, Yale School of Public Health, Yale University, New Haven, CT USA; 3 Department of Public Health Sciences, School of Medicine, University of California, Davis, CA USA; 4 Department of Neurology, and of Epidemiology and Population Health, Albert Einstein College of Medicine, Bronx, NY USA; 5 Departments of Epidemiology and Environmental Health Sciences, School of of Public Health, University of Michigan, Ann Arbor, MI USA

**Keywords:** Coarse particulate matter, Long-term exposure, Inflammation, Coagulation, Women, Menopause

## Abstract

**Background:**

Exposure to particulate matter air pollution has been associated with cardiovascular disease (CVD) morbidity and mortality; however, most studies have focused on fine particulate matter (PM_2.5_) exposure and CVD. Coarse particulate matter (PM_10–2.5_) exposure has not been extensively studied, particularly for long-term exposure, and the biological mechanisms remain uncertain.

**Methods:**

We examined the association between ambient concentrations of PM_10–2.5_ and inflammatory and hemostatic makers that have been linked to CVD. Annual questionnaire and clinical data were obtained from 1694 women (≥ 55 years old in 1999) enrolled in the longitudinal Study of Women’s Health Across the Nation (SWAN) at six study sites from 1999 to 2004. Residential locations and the USEPA air monitoring network measurements were used to assign exposure to one-year PM_10–2.5_, as well as co-pollutants. Linear mixed-effects regression models were used to describe the association between PM_10–2.5_ exposure and markers, including demographic, health and other covariates.

**Results:**

Each interquartile (4 μg/m^3^) increase in one-year PM_10–2.5_ exposure was associated with a 5.5% (95% confidence interval [CI]: 1.8, 9.4%) increase in levels of plasminogen activator inhibitor-1 (PAI-1) and 4.1% (95% CI: − 0.1, 8.6%) increase in high-sensitivity C-creative Protein (hs-CRP). Stratified analyses suggested that the association with PAI-1 was particularly strong in some subgroups, including women who were peri-menopausal, were less educated, had a body mass index lower than 25, and reported low alcohol consumption. The association between PM_10–2.5_ and PAI-1 remained unchanged with adjustment for PM_2.5_, ozone, nitrogen dioxide, and carbon monoxide.

**Conclusions:**

Long-term PM_10–2.5_ exposure may be associated with changes in coagulation independently from PM_2.5_, and thus, contribute to CVD risk in midlife women.

**Supplementary information:**

The online version contains supplementary material available at 10.1186/s12940-020-00663-1.

## Background

Cardiovascular disease (CVD) is the leading cause of death for men and women in the US [1]. Risk factors such as sex, age, increased blood pressure, high cholesterol, and smoking, only account for approximately 50% of cardiac events [2]. Evidence suggests that exposure to particulate matter air pollution is associated with cardiovascular morbidity and mortality, potentially through its ability to increase inflammation and coagulation [3].

Given the known adverse health effects, the United States Environmental Protection Agency (USEPA) regulates particulate matter by setting regulatory standards and enforcing mandatory monitoring for particulate matter with an aerodynamic diameter smaller than 2.5 and 10 μm (PM_2.5_ and PM_10_, respectively) [4]. Most available research has been focused on the health impacts of PM_2.5_ exposure; however, potential health effects from exposure to coarse particulate matter with an aerodynamic diameter between 2.5 and 10 μm (PM_10–2.5_ or coarse particles) might differ from exposure to PM_2.5_ due to the components, sources, and size. Coarse particles are primarily generated by mechanical grinding and resuspension of solid material and may have a high biological content (such as pollen, fungi, and endotoxins) or crustal matter (such as aluminum and silicon and heavy metals) [5]. As a result of size difference, PM_2.5_ deposits deeper in the alveolar region of the lungs, while PM_10–2.5_ mainly deposits higher in the airways [4]. Our understanding of the health impacts from PM_10–2.5_ exposure is limited, and thus, no specific regulatory standards exist for PM_10–2.5_ [4].

Evidence also suggests that PM_10–2.5_ is associated with respiratory and cardiovascular morbidity and mortality [6–11], but the majority of the previous studies have focused on short-term exposure. Powell et al. reported a significant positive association between hospitalizations for CVD and same-day PM_10–2.5_ levels in a Medicare population, aged ≥65 years, in the US [9]. In a systematic review and meta-analysis, respiratory mortality and hospitalizations were increased by 1.4% (95% confidence interval (CI): 0.5–2.4%) and 1.0% (95% CI: 0.1–1.8%), respectively, per 10 μg/m^3^ increase in short-term PM_10–2.5_ exposure [12]. However, the associations between long-term PM_10–2.5_ exposure and mortality/morbidity, which may be derived from different mechanisms from short-term exposure, have been unclear. Early studies reviewed by Brunekreef and Forsberg did not present associations between mortality and morbidity with long-term exposures to PM_10–2.5_ [13]. The meta-analysis study Adar et al. reported a summary estimate of a 2.1% (95% CI: − 1.6 to 5.8%) higher mortality rate per 10 μg/m^3^ increment in long-term PM_10–2.5_ concentration based on six cohort studies, but this association diminished after adjustment for PM_2.5_ [12]. Recent studies examining specific health endpoints have linked long-term exposure to coarse PM with right ventricular dysfunction and hypertension [14, 15].

Furthermore, few studies have assessed associations between PM_10–2.5_ and markers of inflammation and coagulation that are predictive of CVD. Investigating the association between particulate matter and CVD biomarkers could help postulate the underlying physiological mechanisms. Adar et al. found that the endotoxin component of the 5-year concentrations of PM_10–2.5_ was associated with increased inflammation scores, while the copper component of PM_10–2.5_ was associated with an elevated coagulation score [16].

The Study of Women’s Health Across the Nation (SWAN) cohort was designed to follow a multi-racial/ethnic cohort of midlife women through menopausal transition [17]. With longitudinal biomonitoring across years, this study offers a unique opportunity to study CVD risks associated with long-term PM_10–2.5_ exposure. With previous work having shown associations between CVD markers and exposures to ambient PM_2.5_ and gaseous pollutants [18, 19], further research with respect to PM_10–2.5_ exposure and CVD markers in the SWAN cohort would advance the understanding of whether PM_10–2.5_ are associated with inflammation and coagulation. Moreover, with the ability to assess other air pollutants, this study could further evaluate confounding by co-pollutants and improve the understanding of the potential independent impact of PM_10–2.5_ exposure on CVD.

## Methods

### Study population

The SWAN study design and recruitment has been previously described [17]. The present study includes data from six sites: Chicago, Illinois; Detroit, Michigan; Los Angeles, California; Newark, New Jersey; Oakland, California; and Pittsburgh, Pennsylvania. Recruitment included non-Hispanic White women at all sites as well as women from one other racial/ethnic group at each site: African American in Chicago, Pittsburgh, and Detroit; Asian in Oakland (Chinese) and Los Angeles (Japanese); and Hispanic (including women of Central American, Mexican, and Caribbean origin) in Newark. The inclusion criteria for the study at baseline were: being 42 to 52 years of age, having an intact uterus and at least one ovary, no current use of exogenous hormones, not being pregnant or lactating, and having had at least one menstrual period in the last 3 months. Approximately 450 eligible women at each study site have been followed up with annual clinical assessments and interviews.

The present study includes serum samples collected at clinical visits 3 (1999–2000) through visits 7 (2003–2004), since PM_2.5_, a key component for calculating PM_10–2.5_ concentration, was not routinely measured nationwide until 1998.

### CVD marker measurement and analysis

Fasting blood was drawn at each annual SWAN clinical visit and assayed as described previously [18, 20]. The inflammatory and hemostatic markers assessed in this study included high sensitivity C-reactive protein (hs-CRP), fibrinogen, factor VII coagulant (factor VIIc), tissue-type plasminogen activator antigen (tPA-ag), and plasminogen activator inhibitor Type 1 (PAI-1). They serve as markers of the following processes, including systemic inflammation (hs-CRP), formation of blood clots when endothelial damage occurs (fibrinogen and factor VIIc), and fibrinolysis (tPA-ag and PAI-1) [21].

Hs-CRP was measured in serum by immunonephelometry using Behring reagents. Fibrinogen and Factor VII-C were measured in frozen citrated plasma by an assay in which clotting time is measured and compared to a control on a MLA 1400 C coagulation analyzer. PAI-1 and tPA-ag, both free form and tPA-PAI-1 complexes, were measured in plasma using enzyme-linked immunosorbent assay (ELISA) technique (American Diagnostica). A human single chain tPA-ag was used as a standard calibrated against an international standard (National Institute for Biological Standards and Control, Hertfordshire, United Kingdom). PAI-1 was detected using a solid phased monoclonal antibody and a second enzyme-labeled goat antiserum (American Diagnostica) [22]. PAI-1, tPA-ag and hs-CRP were measured at every visit, while fibrinogen and factor VIIc were measured only for visits 3, 5 and 7. All bioassay analyses were conducted in the Medical Research Laboratory, Lexington Kentucky.

Additionally, hs-CRP values > 10 mg/L were excluded from the analyses because they may be indicative of severe infection, major trauma, or chronic inflammatory disease (0.1% of all observations). For the other inflammatory and hemostatic markers, extreme values (outside the mean ± 3 standard deviations after log transformation) were excluded from study (< 3%) as they may indicate laboratory error.

### Exposure assignment

A residential history was obtained for each SWAN participant. Each residence was geocoded, with its coordinate randomly moved up to 400 ft (approximately one block) away to maintain confidentiality. A 20 km circular buffer was created around each address using ArcGIS v10.0 (Environmental Systems Research Institute 2016). The exposure measured by the monitor located in the buffer will be assigned to the participant. For instances in which multiple monitors were located within the buffer, one was chosen based on 1) distance from the residence, and 2) number of visits with exposure data available, which could differ by monitor because of differences in operating dates. Priority was given to the closer monitor to reduce exposure misclassification, but the more distant monitor was chosen if the ratio of (available visits of the distant monitor / available visits of the closer monitor) was greater than the ratio of (the distance to the distant monitor / distance to the closer monitor). If a participant moved between visits, exposure was weighted based on time at each residence using the available move date. If no move date was available, exposure was estimated using the midpoint between the two visits.

Daily PM_10–2.5_ was calculated as the difference of daily PM_2.5_ and PM_10_ concentrations measured at the same monitoring site on the same day, whenever available. Ambient PM_2.5_ and PM_10_ concentrations were obtained from the USEPA air monitoring network, which were monitored every third or sixth day, or daily. The data for these measures were in a 24-h average concentration format. There were fewer monitors for PM_10_, and some monitors take measurements less frequently, which limited the number of measurements of PM_10_, and thus resulting in fewer calculated PM_10–2.5_ measurements. Furthermore, given the uncertainty of extreme values, the top and bottom 2.5% of PM_10_ and of PM_2.5_ data were trimmed.

To assess the potential confounding effects of ambient gases, ozone (O_3_), carbon monoxide (CO), nitrogen dioxide (NO_2_), and sulfur dioxide (SO_2_) concentrations were also obtained. The available data were daily 8-h maximum concentrations for ozone and CO, and one-hour maximum concentrations for NO_2_ and SO_2_. It should be noted that some monitors, specifically in Michigan, only monitored O_3_ during the summer time. All air pollutant data were downloaded from the USEPA Air Data website (https://aqs.epa.gov/aqsweb/documents/data_mart_welcome.html, accessed September 2010).

In this study, we focused on the impact of long-term PM_10–2.5_ exposure, namely the average prior one-year exposure. The six-month exposure was also calculated and considered as intermediate-exposure for a sensitivity analysis. Daily readings were used to calculate average exposure levels for six-month and one-year prior to each blood draw. To simplify, months were considered to be 30-day increments. A minimum of 9 days was necessary for calculating a one-month average; at least 5 months was necessary for a six-month average; and at least 10 months for a one-year average.

### Covariates

The baseline questionnaire, completed when participants were recruited, collected non-time varying covariates on socioeconomic status, for example, residence address, date of birth (for calculating age), race/ethnicity (categorized into White, African American, Asian, or Hispanic), and education (high school or less, some college, or college graduate).

At annual clinic visits, SWAN participants completed questionnaires providing visit-specific information related to medical history, psychosocial environment, lifestyle behaviors, menstrual bleeding patterns, illness, and use of medications since their last visit. Menopausal status was determined based on self-reported bleeding patterns according to standard definitions, and categorized into pre-, early peri-, late peri-, post-menopause, and unknown [23]. Height and weight were measured to calculate body mass index (BMI). Alcohol consumption was divided into three categories developed by Laura L Schott (EDC Coordinating Center): low = none or < 1 serving/month, moderate = up to 1/week or 0.3/day, and high = 2+/week or > 0.3/day.

### Statistical analysis

For each air pollutant and CVD marker, summary statistics were calculated. Biomarker levels were log-transformed to meet the normality assumption. Correlations of markers and air pollutants were calculated based on visit 3 data, as it had the largest sample size among the included visits and serum samples and serves as the baseline for our study.

To study the association between PM_10–2.5_ and CVD biomarkers, we used linear mixed-effects regression models with each biomarker as a continuous, dependent variable. The average prior one-year PM_10–2.5_ exposure was included in a single-pollutant model along with covariates. A random intercept was used to account for covariance of measurements, as multiple longitudinal measurements collected from the same woman are highly correlated. Site was included as a fixed effect because participants were nested within each site. First-order ante-dependence structure was specified for repeated measurements from each participant [19].

Potential covariates were evaluated based on statistical significance and Akaike Information Criterion (AIC) value to control for confounding and goodness of model fit, respectively, without adding over fitted covariates. With all potential covariates, a backward elimination of variables, one at a time, was performed. Variables that have been tested but not included in the final model were: season (cold/warm), physical activity score (continuous), depress symptom score (continuous), poverty score of the ZCTA the participant resided (continuous), antilipidemic medication use (yes/no), and depression medication (yes/no). These variables that were not statistically associated with outcome markers and whose elimination did not change AIC were excluded from the model. The final model included study site, race/ethnicity, education, and visit-specific variables including age (continuous), BMI (continuous), menopausal status, active smoking (yes/no), and alcohol consumption.

Visits after major CVD events, including myocardial infarction, coronary heart failure, stroke, percutaneous coronary intervention, and coronary artery bypass graft, were censored from analyses. We also censored visits for which women did not fast 12 h before blood draw. New Jersey data from visits six and seven were censored because only a few participants had serum sample data for those visits.

Potential effect modification was considered by targeting subgroups for stratification. These groups included BMI (below vs. equal to or above 25 kg/m^2^), current menopause transition stage (early or late peri-menopausal vs. post-menopausal; pre-menopausal was not considered due to small sample size), alcohol consumption category (low vs. medium or high), and education (high school or less vs. some college or more). Each stratified variable was omitted from the base model.

To evaluate the confounding by co-pollutants, we used two-pollutant models, incorporating each of PM_2.5_, ozone, CO, NO_2_, and SO_2_, respectively, along with one-year PM_10–2.5_ in the final models mentioned above, to evaluate the potential confounding effects of each co-pollutant.

Several sensitivity analyses were run excluding women who: had diabetes, were currently smoking, only had one or two visits, with pre-existing medical conditions (including major CVD events defined above, angina, hypertension, or diabetes) and/or medication use, and had unknown menopausal status and/or hormone use.

Analyses were performed in SAS 9.4 (SAS Institute, Cary, NC). All tests were two-sided and *p*-values < 0.05 were considered statistically significant. All results were expressed as the percent change in markers per respective interquartile range increase in air pollutant based on averaging times using the formula [100 × (*exp*^(β per unit pollutant ∗ IQR)^ − 1)].

## Results

After applying the exclusions described above, 1694 women with 5982 observations during the study period were available for analyses. Approximately 68% of the women had three or more clinic visits between 1999 and 2004. The race/ethnicity distribution by study site for this population reflected the SWAN sampling strategy discussed previously. Over 50% of women in Chicago, Los Angeles, and Oakland had completed college, while in Detroit, Newark, and Pittsburgh, approximately 27% of the women had completed college. Most women in the study were overweight (25 < BMI ≤ 30) or obese (BMI ≥ 30), with the exception of women from the Oakland and Los Angeles sites. Additionally, most women in this study were neither current smokers nor high alcohol consumers (2+ servings/week or > 0.3 servings/day) (Table 1).

**Table 1:** Characteristics of the Study Population, SWAN Cohort, 1999–2004

	Detroit, MI	Chicago, IL	Oakland, CA	Los Angeles, CA	Newark, NJ	Pittsburgh, PA
	*n* = 208	*n* = 372	*n* = 343	*n* = 144	*n* = 250	*n* = 377
Race/Ethnicity (%)						
African American	81	52	–	–	–	34
Chinese	–	–	58	–	–	–
Hispanic	–	–	–	–	70	–
Japanese	–	–	–	81	–	–
White	19	48	42	19	30	66
Education (%)						
≤ High school	36	12	19	20	54	23
Some college	42	30	23	29	22	34
≥ College	17	58	58	51	20	43
Total number of visits	804	1420	1454	299	486	1519
Menopausal status (%)						
Pre	4	5	7	5	4	7
Early peri	35	41	37	41	51	34
Late peri	12	10	10	9	11	11
Post	36	37	34	35	26	32
Unknown	12	7	13	10	9	15
Body Mass Index (%)						
< 25, normal	12	20	58	63	22	28
25–30, overweight	23	32	22	23	32	32
> 30, obese	63	40	18	14	39	40
Alcohol consumption (%)						
Low	65	37	60	49	59	49
Medium	17	27	20	22	24	31
High	14	23	18	28	12	19
Current smoker (%)	29	15	3	9	16	14
Diagnosed diabetes (%)	20	8	5	5	10	7
Any CVD event (%)	3.8	0.4	1.2	0	0.4	1.8

Note: percentages do not always add up to 100% because of missing data

The distributions of CVD biomarkers by risk factor status reflect the potential risk factors associated with inflammation and coagulation (Table 2). Women who were current smokers, had diagnosed diabetes, or were obese had higher levels of the inflammatory and hemostatic markers. African American and Hispanic women tended to have higher levels of the inflammatory/ hemostatic markers while Asian women had lower. The two inflammatory markers, hs-CRP and fibrinogen, were moderately correlated (Pearson correlation coefficient (r) = 0.39), as were the two hemostatic markers, PAI-1 and tPA-ag (r = 0.50).

**Table 2:** Distribution of inflammatory and hemostatic biomarkers by demographic factors for SWAN cohort, 1999–2004^a^

Variable		N^b^	hs-CRP^d^	Fibrinogen	Factor VIIc	tPA-ag	PAI-1
(Unit)			mg/l	mg/dl	%	ng/ml	ng/ml
N of samples^c^		5982	4913	2638	2604	5634	5587
All participants		1694	1.6 (3.4)	367.8 (81.0)	130.9 (34.1)	7.1 (4.4)	14.6 (19.5)
Race/Ethnicity	African American	32%	2.7 (4.4)	388.0 (86.4)	130.0 (34.2)	7.9 (4.7)	16.6 (20.2)
	Asian	18%	0.8 (1.3)	354.9 (72.2)	129.0 (28.6)	6.2 (3.9)	11.2 (16.0)
	Hispanic	6%	2.6 (4.0)	370.0 (79.8)	129.1 (34.2)	8.7 (4.3)	20.1 (22.0)
	White	44%	1.6 (3.1)	362.8 (76.8)	134.6 (34.9)	6.9 (4.1)	14.0 (19.7)
	*p-value*^f^		< 0.01	< 0.01	< 0.01	< 0.01	< 0.01
Education	≤ High school	23%	1.7 (3.6)	373.6 (93.1)	134.1 (34.0)	7.4 (4.7)	15.8 (20.3)
	Some college	30%	2.1 (3.7)	373.4 (74.3)	132.7 (33.2)	7.4 (4.4)	16.0 (21.4)
	≥ College	45%	1.4 (3.0)	362.8 (81.6)	130.0 (33.2)	6.7 (4.3)	12.9 (17.8)
	*p-value*		0.03	0.44	0.04	0.11	0.13
Menopausal status	Pre	345	1.6 (2.9)	352.2 (73.2)	129.1 (33.2)	7.1 (4.0)	15.2 (21.2)
	Early peri	2297	1.5 (3.0)	362.4 (81.0)	127.2 (29.7)	7.0 (4.0)	14.5 (18.8)
	Late peri	624	1.6 (3.5)	376.5 (85.0)	134.1 (32.7)	7.7 (4.7)	17.0 (23.2)
	Post	2017	1.8 (3.7)	380.9 (82.5)	136.4 (34.8)	7.4 (4.9)	14.8 (19.4)
	Unknown	687	2.0 (4.0)	362.6 (75.8)	136.0 (37.6)	6.6 (4.1)	12.2 (17.0)
	*p-value*		< 0.01	0.28	< 0.01	< 0.01	< 0.01
Body Mass Index (kg/m^2^)	< 25,	1947	0.8 (1.3)	344.8 (64.1)	123.5 (28.9)	5.4 (3.2)	8.4 (10.2)
	25–30	1672	1.7 (2.6)	365.7 (72.9)	133.7 (34.9)	7.1 (3.6)	14.8 (17.5)
	> 30	2178	3.9 (4.4)	395.3 (82.8)	137.3 (35.7)	8.8 (4.1)	22.6 (23.5)
	*p-value*		< 0.01	< 0.01	< 0.01	< 0.01	< 0.01
Current smoker	Yes	805	2.4 (4.1)	390.9 (87.9)	128.1 (32.4)	7.9 (4.4)	18.7 (23.6)
	No	4959	1.6 (3.1)	364.6 (78.5)	128.1 (32.4)	7.0 (4.3)	13.8 (18.5)
	*p-value*		0.02	< 0.01	0.14	< 0.01	0.04
Alcohol consumption^e^	Low	3084	1.7 (3.7)	375.8 (83.2)	132.7 (33.1)	7.3 (4.6)	15.4 (21.3)
	Moderate	1463	1.7 (3.4)	367.2 (79.3)	130.0 (32.3)	7.0 (4.2)	14.2 (18.1)
	High	1133	1.4 (2.5)	348.9 (75.0)	130.4 (36.0)	6.7 (4.3)	12.2 (17.2)
	*p-value*		0.31	< 0.01	0.18	0.38	0.11
Diagnosed diabetes	Yes	530	4.4 (4.8)	401.1 (88.9)	141.9 (36.3)	9.1 (4.4)	24.4 (27.8)
	No	5449	1.5 (3.1)	365.4 (80.3)	130.9 (32.3)	7.0 (4.3)	13.8 (18.2)
	*p-value*		< 0.01	0.30	< 0.01	< 0.01	< 0.01
Any CVD event	Yes	84	3.4 (4.3)	418.9 (99.1)	145.5 (29.9)	7.1 (4.4)	19.6 (23.7)
	No	5898	1.6 (3.4)	367.1 (80.8)	130.9 (34.1)	8.5 (5.0)	14.5 (19.5)
	*p-value*		0.03	0.08	0.52	0.41	0.25

^a^Data shown in each grid is the median followed by (interquartile range), excluding N

^b^For ethnicity/education, the % show in this column are the percentage of participants in each category among all participants. Percentages do not always add up to 100% because of missing data. For the visit-specific variables, N is the number of observations, not women; each participant could have data from multiple visits and could be in different categories at different visits

^c^Sample size varied by biomarkers. Visits without any blood data or any matched exposure data were excluded. Visits 6 and 7 in New Jersey site were censored due to small sample size. Visits that happened after any CVD events were excluded. Marker values out of reasonable ranges were excluded

^d^For hs-CRP, values > 10 mg/l were not included due to the concern of possible severe inflammation

^e^Alcohol category consists of three categories developed by Laura L Schott (EDC Coordinating Center): low = none or < 1 serving/month, moderate = up to 1/week or 0.3/day, high = 2+/week or > 0.3/day

^f^*p-value* are from the Type 3 test of fixed effects using mixed effect model, with all variables included as fixed effects and a random intercept account for covariance of measurements. Site was also included as a fixed effect and participants were nested within each site, as multiple longitudinal measurements collected from the same woman are highly correlated. First-order ante-dependence structure was specified for repeated measurements from each participant

The average prior one-year PM_10–2.5_ exposures for all the SWAN sites were 10.9 ± 3.6 μg/m^3^ (Table 3). The average prior six-month exposure was similar (10.8 ± 4.0 μg/m^3^), and had a slightly larger sample size (*N* = 5466) than the one-year exposure (*N* = 5175), because PM_2.5_ measurements started from 1998 which limited the ability to calculate prior one-year exposure for some early visits. Los Angeles site had the highest levels of one-year PM_10–2.5_ exposure at 17.5 ± 2.3 μg/m^3^, while Pittsburgh had the lowest levels at 8.5 ± 3.8 μg/m^3^. Due to the limited availability of PM_10_ monitors, however, Los Angeles only had a small number of visits with co-located PM_10_ and PM_2.5_ monitors, and thus matched PM_10–2.5_ data. Distributions of one-year average exposure of co-pollutants, including PM_2.5_, ozone, CO, NO_2_ and SO_2_ exposure, can be found in Table S1.

**Table 3:** Descriptive statistics for prior one-year exposure to coarse particulate matter (PM_10–2.5_) concentrations (μg/m^3^) for SWAN sites, 1999–2004

Site	N	Mean	SD	Median	IQR
All sites	5175	10.9	3.6	11.5	4.0
By site					
Detroit, MI	719	10.6	3.5	10.3	3.9
Chicago, IL	1296	12.1	1.9	12.0	2.4
Oakland, CA	1229	10.9	1.8	11.6	2.2
Los Angeles, CA	209	17.5	2.3	18.4	1.6
Newark, NJ	366	12.4	4.9	12.8	5.7
Pittsburgh, PA	1356	8.5	3.8	7.4	6.8

We observed that an interquartile (4 μg/m^3^) increase in the prior one-year exposure of PM_10–2.5_ was associated with a 5.5% (1.8, 9.4%) increase in PAI-1 level. This association was consistent for the six-month exposure window, with a 4.0% (0.3, 7.8%) increase in PAI-1 per 4 μg/m^3^ increase in PM_10–2.5_. The inflammation marker, hs-CRP, was also associated with PM_10–2.5_, with a 4.1% (− 0.1, 8.6%) and a 6.0% (1.7, 10.5%) increase in hs-CRP per 4 μg/m^3^ increase in prior one-year and six-month PM_10–2.5_ exposure, respectively. Associations with other markers were not observed.

Sensitivity analyses were conducted with some subgroups removed. Positive associations between PM_10–2.5_ and PAI-1 levels remained statistically significant in models that excluded women with diagnosed diabetes, those who reported current smoking, who did not complete at least three clinic visits, who reported medical preconditions, who reported medication use, and who had unknown menopausal status and hormone use, respectively (Table S2). The marginal association between PM_10–2.5_ and hs-CRP became statistically significant in the model restricting analysis to women who completed three or more visits and in the model excluding smokers; however, the association disappeared in other sensitivity analyses.

Potential effect modifiers were evaluated for the one-year PM_10–2.5_ exposure, and some subgroups appeared to have experienced elevated risks (Table 4). For a 4 μg/m^3^ increase in one-year PM_10–2.5_, PAI-1 increased by 9.2% (1.4, 17.7%) in women with a BMI <  25, 7.5% (2.2, 13.2%) in peri-menopausal women, 7.6% (0.6, 15.1%) in women with equivalent or less than a high school education, and 7.5% (2.3, 13.0%) in women who reported low alcohol consumption. Additionally, women who reported low alcohol consumption had an 8.8% (2.7, 15.3%) increase in hs-CRP, and women with equivalent or less than a high school education had 2.4% (0.1, 4.7%) decrease in factor VIIc, per 4 μg/m^3^ increase in PM_10–2.5_. The stratification results for the other inflammatory and hemostatic markers were not statistically significant.

**Table 4:** Adjusted associations between PM_10–2.5_ and cardiovascular disease markers for SWAN cohort, 1999–2004^a^

Marker	hs-CRP	Fibrinogen	Factor VIIc	tPA-ag	PAI-1
Total	4.1 (−0.1, 8.6)*	−0.1 (−1.0, 0.8)	−0.1 (−1.2, 1.1)	0.4 (− 1.4, 2.3)	5.5 (1.8, 9.4)***
BMI < 25	5.4 (−3.1, 14.5)	Did not converge	0.6 (− 1.5, 2.7)	2.9 (− 1, 7.0)	9.2 (1.4, 17.7)**
BMI ≥ 25	4.5 (− 0.3, 9.5)*	− 0.02 (− 1.1, 1.1)	− 0.8 (−2.2, 0.7)	−1.3 (− 3.3, 0.8)	3.3 (− 0.9, 7.6)
Peri-menopausal	1.8 (−4.4, 8.4)	− 0.1 (− 1.4, 1.3)	− 1.4 (− 2.9, 0.2)*	0.05 (− 2.4, 2.5)	7.5 (2.2, 13.2)***
Post-menopausal	3.6 (−2.8, 10.5)	−0.02 (− 1.1, 1.1)	0.1 (− 1.9, 2.2)	0.3 (− 2.8, 3.6)	1.4 (−4.5, 7.7)
≤ High school	5.8 (−2.7, 15)	1.2 (− 0.7, 3.2)	−2.4 (− 4.7, − 0.1)**	0.9 (− 2.6, 4.5)	7.6 (0.6, 15.1)**
Some college or more	3.8 (−1.1, 8.9)	− 0.9 (− 1.9, 0.2)	0.05 (− 1.3, 1.4)	0.3 (−1.8, 2.5)	1.1 (− 3.2, 5.6)
Low alcohol consumption	8.8 (2.7, 15.3)***	0.6 (− 0.7, 1.8)	−1.4 (− 3.0, 0.2)*	1.3 (− 1.2, 3.9)	7.5 (2.3, 13.0)***
Medium/high alcohol consumption	−0.2 (− 6.1, 6.0)	−1.2 (− 2.5, 0.2)*	0.5 (− 1.2, 2.2)	− 0.9 (− 3.6, 1.8)	3.9 (−1.4, 9.5)

^a^Results shown are as percent change in biomarker level per an interquartile increase in exposure, which is 4 μg/m^3^ for PM_10–2.5_. Analyses were based on log-transformed biomarker levels, *adjusted for study site, age (continuous), race/ethnicity, education, menopausal status, BMI, active smoking status, alcohol category. Stratifying variables were omitted from respective models.* *p* < 0.10; ** *p* < 0.05; *** *p* < 0.01

The co-pollutant models were conducted in subsets of observations compared with the single pollutant models of PM_10–2.5_, given the availability of co-pollutant data. For most sites, 70–98% of serum samples had matched co-pollutant exposure, except for ozone in Detroit (0.5%) (not included in the analysis) and Newark (48%), NO_2_ in Detroit (57%), Chicago (65%), and Newark (65%), and SO_2_ in Oakland (53%) (Table S1). One-year PM_10–2.5_ exposure appeared to be moderately correlated with one-year PM_2.5_ (*r* = 0.29) and NO_2_ (*r* = 0.30). The interquartile ranges for the prior one-year average exposures of co-pollutants was 3 μg/m^3^ for PM_2.5_, 0.007 ppm for ozone, 0.3 ppm for CO, 5 ppb for NO_2_, and 3.0 ppb for SO_2_.

**Fig. 1:**
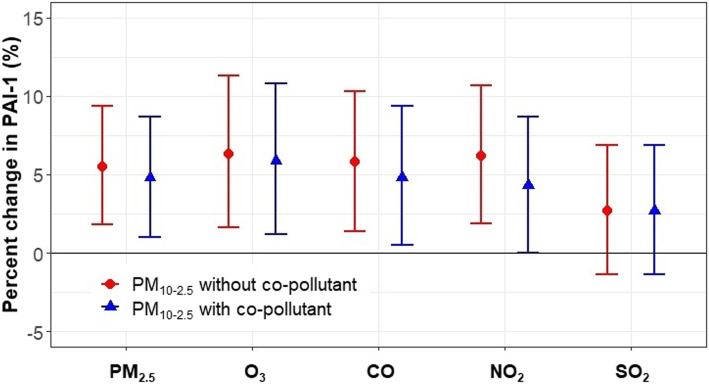
Associations between PM_10–2.5_ and PAI-1, without (dot symbol) and with (triangle symbol) PM_2.5_, O3, CO, NO_2_, and SO_2_ adjusted in the SWAN cohort, 1999–2004. Note: Results shown are percent of change in biomarker level per an interquartile increase of PM_10–2.5_ exposure, which is 4 μg/m^3^. Analyses were based on log-transformed biomarker levels, adjusted for study site, age (continuous), race/ethnicity, education, menopausal status, BMI, active smoking status, alcohol consumption category. For each co-pollutant, analyses were run in the subset with non-missing values for both PM_10-2.5_ and the co-pollutant

Our previous studies have found that one-year exposures to PM_2.5_, NO_2_, and CO were positively associated with PAI-1 [18, 19]. In two-pollutant models along with PM_10–2.5_, PM_2.5_, CO, and NO_2_ were each still positively associated with PAI-1 levels, and the associations were stronger for PM_2.5_, CO, and NO_2_ than PM_10–2.5_. In contrast, O_3_ had a negative association with PAI-1. Nevertheless, these co-pollutants did not confound the association between PM_10–2.5_ and PAI-1 (Fig. 1 and Table S3). Specifically, the positive association remained for PAI-1 and PM_10–2.5_ from the single pollutant model when considering co-exposure to PM_2.5_, with a 4.8% (1.0, 8.7%) increase per 4 μg/m^3^ increase in PM_10–2.5_. Similarly, PAI-1 increased by 4.8% (0.5, 9.4%) per 4 μg/m^3^ increase in PM_10–2.5_ after adjustment for co-exposure to CO, and by 4.3% (0, 8.7%) per 4 μg/m^3^ increase in PM_10–2.5_ independent of exposure to NO_2_. Confounding by SO_2_ cannot be evaluated, because in the subset of data with matched SO_2_ measurements, PM_10–2.5_ showed no association with PAI-1 regardless of including SO_2_ or not. The availability of SO_2_ measurements may have introduced bias for testing the association between PM_10–2.5_ and PAI-1.

## Discussion

This study provided new evidence for associations between long-term exposure to coarse particles and CVD markers. Specifically, we found that prior one-year PM_10–2.5_ exposure levels were positively associated with the levels of PAI-1, and marginally positively associated with hs-CRP. Elevated PAI-1 increases the risk of thrombosis by inhibiting tPA activity, which initiates the fibrinolysis processes that break down excess blood clotting. Therefore, the associations we found suggested that elevated long-term exposure to coarse particles may increase the risk of thrombosis.

As mentioned earlier, very few studies were conducted on the associations of long-term exposure to PM_10–2.5_ with CVD markers. Chen and Schwarz observed increased white blood cell count, a potential inflammatory marker, was associated with one-year local PM_10_ levels (ranging 14.6–78.5 μg/m^3^) in the US [24]. In contrast, in England, Forbes et al. reported no associations between inflammatory markers, fibrinogen or hs-CRP, and chronic exposures to several outdoor pollutants, including PM_10_ (ranging 11.0–36.1 μg/m^3^) [25]. Inconsistencies in study findings could have resulted from differences in air pollutant ranges, geographic and temporal differences in the composition of particles, and/or population differences. The Multi-Ethnic Study of Atherosclerosis estimated 5-year average PM_10–2.5_ exposures between 2000 and 2006 varied by individuals, with a mean of 5.0 ± 1.7 μg/m^3^, and found positive, though not statistically significant, associations between long-term PM_10–2.5_ concentrations and inflammation and coagulation markers [16]. The authors further identified that endotoxin and copper in PM_10–2.5_ contributed most to inflammation and coagulation effects, respectively, which were robust to the adjustment for PM_2.5_. As an indicator of abrasive brake wear, copper is expected in substantial amount in PM_10–2.5_ at most of the SWAN sites, which are major metropolitan areas with intense traffic flow, particularly in Los Angeles [26]. Our study echoes Adar et al’s findings, providing additional evidence on the impact of PM_10–2.5_ coagulation among different locations and populations.

Our previous studies have identified associations between exposures to PM_2.5_ and ambient gases with inflammatory and hemostatic markers in the SWAN cohort. One-year exposure to PM_2.5_ was associated with increases in hs-CRP (% of change [95th% confidence interval]: 21% [6.6, 37%]) and PAI-1 (35% [19, 53%]) per 10 μg/m^3^ increase in PM_2.5_ concentrations [18]. One-year exposure to CO and NO_2_ were also positively associated with PAI-1, though not statistically significant [19]. We note that, while the increased inflammation and coagulation tendency associated with PM_10–2.5_ was similar to that from PM_2.5_, PM_10–2.5_ exposure was weakly correlated with PM_2.5_, regardless of whether long-term (Spearman r = 0.30, *p* < 0.01) or short-term (*r* = 0.15, *p* < 0.01) exposure. The two-pollutant analyses confirmed the associations with PM_2.5_ and ambient gases, and further demonstrated that PM_10–2.5_ created additional burden on inflammation and coagulation. The previous studies did not control for PM_10–2.5_, so some of the associations observed may have been due to confounding by PM_10–2.5_.

Considering the variations in chemical composition and the deposition locations within the lungs associated with their sizes, we expect that PM_10–2.5_ cause health effects through mechanisms different from those of PM_2.5_. However, it has been observed that coarse PM of low density, such as soil particles, have deposited deeper in the lung as well [27], suggesting that they could cause potential health impacts beyond size alone. A study using in vitro models found out that PM_2.5_ and PM_10–2.5_ affected nasal and bronchial epithelial cells and immune response differently, but both increased release of IL-6 in bronchial epithelial cells [28]. Ljubimova et al. observed up regulation of genes encoding inflammatory cytokine pathways (IL13-Rα1 and IL-16) in the brains of rats exposed to PM_10–2.5_ sourced from the Los Angeles Basin for 1 month, and found that PM_10–2.5_ was the only particles contributing to this process [29]. A few toxicological studies has indicated that PM_10–2.5_ may have stronger associations with inflammation and coagulation than PM_2.5_ [30–34].

Furthermore, risks derived from short-term and long-term exposure may be generated through different mechanisms. Coagulation activation, indicated by the increase of PAI-1 level, is a typical risk that has been associated with long-term exposure [35]. Two potential mechanisms could explain the increased PAI-1. One possibility is that, some PM_10–2.5_ particles travel deeper in the lung and cause systemic inflammation and oxidative stress similar to that from PM_2.5_. Generated by the endothelium, PAI-1 is activated by chronic inflammatory conditions and endothelium injury to inhibit fibrinolysis, the process that degrades blood clots; in other words, PAI-1 allows blood clot formation and shortens bleeding time. Another possible pathway is that PM_10–2.5_ induces respiratory tract inflammation. Inflammatory cytokines produced in the respiratory tract can potentially enter the circulatory system where they can stimulate the liver to release coagulation factors that can alter hemostasis [4]. This could be a “false” coagulation signal that is not accompanied by systemic inflammation or blood vessel damage that initiates fibrinolysis, eventually increasing the potential for thrombosis. Under such circumstances, there may not be clear signs of systemic inflammation and/or endothelium injury. Overall, evidence from epidemiologic or toxicological studies is limited for systemic inflammation and altered hemostasis associated with the long-term exposure to PM_10–2.5_. Given the marginal/unstable association with hs-CRP and absent relationship with fibrinogen and factor VIIc, we cannot conclude whether inflammation and thrombosis were from the same biological process, or differentiate whether such coagulation reflected vascular inflammation or deep vein thrombosis. Future studies are warranted to explore the physiological consequences and mechanisms involved with chronic exposure to PM_10–2.5_, especially to the specific chemical species of PM_10–2.5_.

Our findings also suggest that the associations between PAI-1 and one-year PM_10–2.5_ exposure appeared particularly strong in some subgroups, including women with a BMI <  25, women who were peri-menopausal, women with equivalent or less than a high school education, and women who reported low alcohol consumption. That is to say, apart from toxicity of chemicals, physiological condition, i.e., BMI and menopausal status, and socioeconomic background, i.e., education, could influence vulnerability to external exposures. For women, the menopausal transition is a complex and multifaceted process that involves multiple organ systems and genetic variability where physiological impacts can manifest differently among individual women [17]. Being in midlife and experiencing a transition through menopause potentially increases women’s vulnerability to environmental exposures, such as air pollution.

This study had several strengths. First, SWAN is a large, multi-site, longitudinal study, which provided coverage of a wide range of ambient exposure levels and particle compositions associated with local sources. Also, with the same participants followed longitudinally and the same sampling/analytical approaches for measurement of biomarkers consistently used, our data provided a unique opportunity to examine health effects associated with long-term PM_10–2.5_ exposure. Second, because residential history has been maintained for each SWAN participant, we were able to use ambient monitoring data, along with residential history, to assign exposure levels to participants. Finally, substantial demographic and health information collected in SWAN allowed us to examine the impact of effect modifiers, including time-varying menopausal status for this midlife population.

Meanwhile, the limitations of estimating PM_10–2.5_ exposure need to be noted when interpreting our results. PM_10–2.5_ was not routinely measured directly, its level was either calculated through the ambient monitor concentrations of PM_10_ and PM_2.5_ as we did, or obtained by modeling. Some studies used estimations from land use regression models, which were based on limited cross-sectional measurements to simulate long-term spatial variation [14, 16]. The 5-year average PM_10–2.5_ exposures of the Multi-Ethnic Study of Atherosclerosis cohort were estimated using land use regression model, ranging from 3.8 ± 1.3 μg/m^3^ in Winston-Salem, North Carolina to 5.6 ± 1.2 μg/m^3^ in Chicago, Illinois [16]. Models based on satellite data are not applicable to this study, as most satellite data are only available after early 2000, and therefore, would not cover a significant amount of SWAN visits around 2000. Furthermore, PM_10–2.5_ concentrations were not commonly estimated through satellite data in the USA, as PM_2.5_ has been the primary focus. We acknowledge that PM_10–2.5_ concentration tends to be more heterogeneous across space than PM_2.5_, resulting in great uncertainty for PM_10–2.5_ estimations through nearby stationary monitors within a 20 km buffer. The assigned exposure may be overestimated or underestimated depending on whether there are sources nearby. We assume that the exposure assignment error tends to be non-differential [36]. Additionally, in this study, we calculated PM_10–2.5_ concentrations using data obtained from co-located PM_10_ and PM_2.5_ monitors to avoid potential bias. However, PM_10_ monitoring had been greatly reduced (typically every 6 days for PM_10_, compared to typically every 3 days for PM_2.5_), which limited the ability to calculate PM_10–2.5_ data. This was a particular concern for the Los Angeles site, where only 24% of women had matched PM_10–2.5_ measurements. The mean average one-year PM_10–2.5_ exposure in our study ranged from 8.5 ± 3.8 μg/ m^3^ to 17.5 ± 2.3 μg/m^3^ at different sites, which aligned with the levels reported in the literature.

A few other limitations also need to be considered. First, as a common issue in cohort studies, loss of follow-up was also observed in SWAN, which reduced statistical power. In our dataset, 35% of participants had completed one or two visits only, including almost all participants at Newark site and half of the participants at Los Angeles site, which was equivalent to 20% of visits. We were not able to run the regression model within these subjects because of the small sample size; however, sensitivity tests excluding these participants confirmed the associations we observed (Table S2), including those from stratified analyses (not shown). Second, potential confounding factors, such as, noise, greenness, household income, were not included in this analysis due to limited available data. Future studies are recommended to include them into considerations. Lastly, heterogeneity of PM_10–2.5_ compositions by study location may influence the associations with biomarkers, but composition information was not available to use in our study.

## Conclusions

The results of this study support the hypothesis that long-term exposure to coarse particulate matter may contribute to inflammation and thrombosis, markers of CVD. The observed associations between long-term PM_10–2.5_ exposure and PAI-1 were independent, as they were not confounded by ambient PM_2.5_, ozone, NO_2_, or CO. Particular subgroups were more sensitive to PM_10–2.5_ exposure. Further epidemiological and toxicological studies are warranted to identify the specific mechanisms of how PM_10–2.5_ may affect these biomarkers.

## Supplementary information

Additional file 1: **Table S1.** Distribution of prior one-year average PM_2.5_, ozone, CO, NO_2_, and SO_2_ concentrations by SWAN site, 1999–2004. **Table S2**. Results of sensitivity test for the associations between PM_10–2.5_ and cardiovascular disease markers based on several models for SWAN cohort, 1999–2004. **Table S3**. Adjusted associations between PM_10–2.5_ and PM_2.5_, Ozone, CO, NO_2_, SO_2_ and cardiovascular disease markers for SWAN cohort, 1999–2004. (docx 29 kb)

## Data Availability

The data that support the findings of this study are available from California Office of Statewide Health Planning and Development (OSHPD) but restrictions apply to the availability of these data, which were used under license for the current study, and so are not publicly available. Data are however available from the authors upon reasonable request and with permission of OSHPD.

## Abbreviations

CVD: Cardiovascular disease
PM_2.5_: Fine particulate matter
PM_10–2.5_: Coarse particulate matter
SWAN: Study of Women’s Health Across the Nation
US EPA: United States Environmental Protection Agency
hs-CRP: High sensitivity C-reactive protein
factor VIIc: Factor VII coagulant
tPA-ag: Tissue-type plasminogen activator antigen
PAI-1: Plasminogen activator inhibitor Type 1
ELISA: Enzyme-linked immunosorbent assay
O_3_: Ozone
CO: Carbon monoxide
NO_2_: Nitrogen dioxide
SO_2_: Sulfur dioxide
BMI: Body mass index
AIC: Akaike Information Criterion
ZCTA: ZIP code tabulation area
