# Supplementary material for: Association between coarse particulate matter and inflammatory and hemostatic markers in a cohort of midlife women
Source: Environ Health. 2020 Nov 5;19:111. doi: 10.1186/s12940-020-00663-1 (PMC7643259; doi:10.1186/s12940-020-00663-1)
Supplement: Supplementary file 1 — Table S1. Distribution of prior one-year average PM2.5, ozone, CO, NO2, and SO2 concentrations by SWAN site, 1999–2004. Table S2. Results of sensitivity test for the associations between PM10–2.5 and cardiovascular disease markers based on several models for SWAN cohort, 1999–2004. Table S3. Adjusted associations between PM10–2.5 and PM2.5, Ozone, CO, NO2, SO2 and cardiovascular disease markers for SWAN cohort, 1999–2004. (docx 29 kb) [file 12940_2020_663_MOESM1_ESM.docx]

Table S1. Distribution of prior one-year average PM_2.5_, ozone, CO, NO₂, and SO₂ concentrations by SWAN site, 1999–2004.

|  |  | 1-year average concentration | | | | | |
| --- | --- | --- | --- | --- | --- | --- | --- |
| Site |  | N | %* | Mean | SD | Median | IQR |
| PM2.5 (µg/m³) |  |  |  |  |  |  |  |
| Detroit, MI |  | 751 | 93 | 16.3 | 1.6 | 16.0 | 1.6 |
| Chicago, IL |  | 1265 | 89 | 16.1 | 1.2 | 16.2 | 1.9 |
| Oakland, CA |  | 890 | 61 | 12.0 | 1.3 | 12.0 | 1.7 |
| Los Angeles, CA |  | 290 | 97 | 20.3 | 2.4 | 20.6 | 2.7 |
| Newark, NJ |  | 400 | 82 | 16.1 | 1.3 | 16.4 | 1.7 |
| Pittsburgh, PA |  | 1352 | 89 | 16.1 | 1.7 | 16.1 | 1.9 |
|  |  |  |  |  |  |  |  |
| Ozone (ppm) |  |  |  |  |  |  |  |
| Detroit, MI |  | 4 | 0.5 | 0.026 | 0.001 | 0.027 | 0.001 |
| Chicago, IL |  | 1321 | 93 | 0.020 | 0.001 | 0.019 | 0.002 |
| Oakland, CA |  | 1126 | 77 | 0.018 | 0.004 | 0.024 | 0.007 |
| Los Angeles, CA |  | 267 | 89 | 0.025 | 0.004 | 0.024 | 0.007 |
| Newark, NJ |  | 231 | 48 | 0.027 | 0.006 | 0.029 | 0.008 |
| Pittsburgh, PA |  | 1390 | 92 | 0.023 | 0.002 | 0.023 | 0.002 |
|  |  |  |  |  |  |  |  |
| CO (ppm) |  |  |  |  |  |  |  |
| Detroit, MI |  | 665 | 83 | 0.36 | 0.08 | 0.34 | 0.08 |
| Chicago, IL |  | 1256 | 88 | 0.76 | 0.15 | 0.78 | 0.27 |
| Oakland, CA |  | 1346 | 93 | 0.66 | 0.13 | 0.67 | 0.21 |
| Los Angeles, CA |  | 264 | 88 | 0.65 | 0.20 | 0.56 | 0.28 |
| Newark, NJ |  | 361 | 74 | 1.21 | 0.30 | 1.16 | 0.37 |
| Pittsburgh, PA |  | 1156 | 76 | 0.68 | 0.28 | 0.64 | 0.56 |
|  |  |  |  |  |  |  |  |
| NO₂ (ppb) |  |  |  |  |  |  |  |
| Detroit, MI |  | 462 | 57 | 22.4 | 1.8 | 22.1 | 3.3 |
| Chicago, IL |  | 928 | 65 | 23.2 | 1.7 | 23.0 | 1.9 |
| Oakland, CA |  | 1294 | 89 | 19.1 | 2.1 | 19.3 | 2.5 |
| Los Angeles, CA |  | 255 | 85 | 25.2 | 3.3 | 24.6 | 3.7 |
| Newark, NJ |  | 318 | 65 | 34.1 | 5.8 | 36.4 | 11.2 |
| Pittsburgh, PA |  | 1483 | 98 | 21.2 | 2.4 | 21.2 | 2.4 |
|  |  |  |  |  |  |  |  |
| SO₂ (ppb) |  |  |  |  |  |  |  |
| Detroit, MI |  | 588 | 73 | 6.4 | 1.2 | 6.3 | 2.0 |
| Chicago, IL |  | 1343 | 95 | 6.2 | 3.0 | 5.1 | 5.2 |
| Oakland, CA |  | 771 | 53 | 1.8 | 0.5 | 1.8 | 0.8 |
| Los Angeles, CA |  | 233 | 78 | 2.2 | 1.4 | 2.5 | 2.8 |
| Newark, NJ |  | 414 | 85 | 9.5 | 2.6 | 8.9 | 5.0 |
| Pittsburgh, PA |  | 1213 | 80 | 9.2 | 1.6 | 9.5 | 2.2 |
|  |  |  |  |  |  |  |  |

*Percentages of blood samples with geographically matched particulate matter and gas concentrations from monitors within 20 km buffer of participants' residences.

Table S2. Results of sensitivity test for the associations between PM_10-2.5_ and cardiovascular disease markers based on several models for SWAN cohort, 1999–2004.

| Marker | hs-CRP | Fibrinogen | Factor VIIc | tPA-ag | PAI-1 |  |
| --- | --- | --- | --- | --- | --- | --- |
|  |  |  |  |  |  |  |
| Base | 4.1 (-0.1 , 8.6)* | -0.1 (-1.0 , 0.8) | -0.1 (-1.2 , 1.1) | 0.4 (-1.4 , 2.3) | 5.5 (1.8 , 9.4)*** |  |
| Model 1 | 3.5 (-0.9 , 8.2) | -0.4 (-1.4 , 0.6) | -0.2 (-1.5 , 1.0) | 0.4 (-1.6 , 2.3) | 6.5 (2.5 , 10.8)*** |  |
| Model 2 | 5.2 (0.5 , 10.1)** | -0.2 (-1.2 , 0.9) | -0.5 (-1.8 , 0.8) | 0.8 (-1.2 , 2.9) | 6.1 (1.9 , 10.5)*** |  |
| Model 3 | 4.9 (0.2 , 9.8)** | -0.3 (-1.3 , 0.7) | -0.4 (-1.7 , 0.9) | 0.5 (-1.5 , 2.6) | 5.4 (1.2 , 9.8)** |  |
| Model 4 | 3.8 (-1.9 , 9.9) | 0.05 (-1.2 , 1.3) | -0.5 (-2.0 , 1.0) | 1.1 (-1.4 , 3.7) | 8.1 (2.8 ,13.6)*** |  |
| Model 5 | 3.8 (-1.9 , 9.9) | 0.05 (-1.2 , 1.3) | -0.5 (-2.0 , 1.0) | -0.2 (-2.3 , 1.9) | 5.0 (0.8 , 9.4)** |  |
| Model 6 | 3.5 (-1.3 , 8.5) | -0.5 (-1.5 , 0.6) | -1.6 (-2.9 , -0.3)** | 0.2 (-1.8 , 2.2) | 5.4 (1.2 , 9.8)** |  |
|  |  |  |  |  |  |  |

Note: Results shown are percent of change in biomarker level per an interquartile increase of exposure, which is 4 µg/m³ for PM_10-2.5_.

Base: Analyses were based on log-transformed biomarker levels, adjusted for study site, age (continuous), race/ethnicity, education, menopause status, BMI, active smoking status, alcohol category.

Model 1: Base, excluding diagnosed diabetes.

Model 2: Base, excluding smokers.

Model 3: Base, inclusion of women who participated in 3 or more clinic visits.

Model 4: Base, excluding women with medical preconditions.

Model 5: Base, excluding clinic visits with reported medication use.

Model 6: Base, excluding women with unknown menopause status or post-menopausal by Bilateral Salpingo Oophorectomy or clininc visits with reported hormone use

p < 0.10; ** p < 0.05; *** p < 0.01.

Table S3. Adjusted associations between PM_10-2.5_ and PM_2.5_, Ozone, CO, NO₂, SO₂ and cardiovascular disease markers for SWAN cohort, 1999–2004.

|  |  | PM_10-2.5_ only model | PM_10-2.5_ with co-pollutant | |
| --- | --- | --- | --- | --- |
| **effect** |  | PM_10-2.5_ | PM_10-2.5_ | Co-pollutant |
|  |  |  |  | **PM_2.5_** |
| Subset with no-missing PM_2.5_ | hs-CRP | 4.1 (-0.1 , 8.6)* | 3.2 (-1.1 , 7.7) | 4.5 (-1.7 , 11.1) |
|  | Fibrinogen | -0.1 (-1.0 , 0.8) | -0.3 (-1.2 , 0.6) | 0.2 (-1.2 , 1.5) |
|  | Factor VIIc | -0.1 (-1.2 , 1.1) | -0.3 (-1.5 , 0.9) | -1.4 (-3.1 , 0.3)* |
|  | tPA-ag | 0.4 (-1.4 , 2.3) | 0.2 (-1.7 , 2.1) | -0.3 (-3 , 2.5) |
|  | PAI-1 | 5.5 (1.8 , 9.4)*** | 4.8 (1.0 , 8.7)** | 6 (0.5 , 11.8)** |
|  |  |  |  | **ozone** |
| Subset with no-missing ozone | hs-CRP | 3.1 (-2 , 8.4) | 3 (-2.1 , 8.4) | -1.5 (-9.6 , 7.5) |
|  | Fibrinogen | -1.1 (-2.2 , 0)* | -1.1 (-2.2 , 0)* | -0.04 (-2.02 , 1.98) |
|  | Factor VIIc | 0.1 (-1.3 , 1.5) | 0.5 (-0.9 , 1.9) | 5.9 (3.3 , 8.5)*** |
|  | tPA-ag | 0.4 (-1.9 , 2.7) | 0.4 (-1.8 , 2.7) | 0.9 (-2.9 , 4.8) |
|  | PAI-1 | 6.3 (1.6 , 11.3)*** | 5.9 (1.2 , 10.8)** | -7.8 (-14.8 , -0.3)** |
|  |  |  |  | **CO** |
| Subset with no-missing CO | hs-CRP | 3.3 (-1.6 , 8.4) | 3.7 (-1.3 , 8.9) | -3.5 (-8.9 , 2.1) |
|  | Fibrinogen | 0.1 (-0.9 , 1.1) | 0.03 (-0.98 , 1.05) | 0.3 (-0.8 , 1.5) |
|  | Factor VIIc | 0.1 (-1.2 , 1.4) | 0.1 (-1.2 , 1.4) | -0.2 (-1.7 , 1.3) |
|  | tPA-ag | 0.5 (-1.6 , 2.6) | 0.2 (-1.9 , 2.3) | 2.4 (-0.1 , 4.8)* |
|  | PAI-1 | 5.8 (1.4 , 10.3)*** | 4.8 (0.5 , 9.4)** | 7.5 (2.4 , 13)*** |
|  |  |  |  | **NO_2_** |
| Subset with no-missing NO_2_ | hs-CRP | 5.3 (0.4 , 10.4)** | 4.6 (-0.3 , 9.7)* | 7.7 (0.1 , 15.8)** |
|  | Fibrinogen | -0.8 (-1.9 , 0.2) | -0.8 (-1.9 , 0.3) | 0.02 (-1.66 , 1.74) |
|  | Factor VIIc | 0.3 (-1.1 , 1.7) | 0.4 (-1 , 1.7) | -0.4 (-2.6 , 1.8) |
|  | tPA-ag | 1.2 (-0.9 , 3.3) | 1.3 (-0.8 , 3.4) | -1.1 (-4.3 , 2.2) |
|  | PAI-1 | 6.2 (1.9 , 10.7)*** | 4.3 (0 , 8.7)** | 17.8 (10.4 , 25.6)*** |
|  |  |  |  | **SO_2_** |
| Subset with no-missing SO_2_ | hs-CRP | 4.4 (-0.3 , 9.4)* | 4.3 (-0.4 , 9.3)* | 1.9 (-2.5 , 6.5) |
|  | Fibrinogen | 0.1 (-1 , 1.1) | 0.1 (-0.9 , 1.1) | -0.4 (-1.5 , 0.6) |
|  | Factor VIIc | 0.6 (-0.7 , 1.9) | 0.6 (-0.7 , 1.9) | -0.3 (-1.6 , 1) |
|  | tPA-ag | 0.3 (-1.7 , 2.4) | 0.3 (-1.8 , 2.4) | 0.2 (-1.7 , 2.1) |
|  | PAI-1 | 2.7 (-1.4 , 6.9) | 2.7 (-1.4 , 6.9) | 0.3 (-3.5 , 4.3) |
|  |  |  |  |  |

Note: Results shown are percent of change in prior one-year biomarker level per an interquartile increase of exposure, which is 4 µg/m³ for PM_10-2.5_, 3 µg/m³ for PM_2.5_, 0.007 ppm for Ozone, 0.3 ppm for CO, 5 ppb for NO₂, and 3 ppb for SO₂. Analyses were based on log-transformed biomarker levels, adjusted for study site, age (continuous), race/ethnicity, education, menopause status, BMI, active smoking status, alcohol category. p < 0.10; ** p < 0.05; *** p < 0.01.
